# Accuracy of automatic matching of Catphan 504 phantom in cone‐beam computed tomography for tube current–exposure time product

**DOI:** 10.1120/jacmp.v17i6.6402

**Published:** 2016-11-08

**Authors:** Hui‐Su Won, Jin‐Beom Chung, Byung‐Don Choi, Jin‐Hong Park, Do‐Guwn Hwang

**Affiliations:** ^1^ Department of Biomedical Engineering Sangji University Wonju South Korea; ^2^ Department of Radiation Oncology Seoul National University Bundang Hospital Seongnam South Korea; ^3^ Department of Radiological Technology Shingu University Seongnam South Korea

**Keywords:** cone‐beam computed tomography, mAs, image quality, automatic matching

## Abstract

The purpose of this study is to evaluate the accuracy of automatic matching in cone‐beam computed tomography (CBCT) images relative to the reduction of total tube current–exposure time product (mAs) for the X‐ray imaging (XI) system. The CBCT images were acquired with the Catphan 504 phantom various total mAs ratios such as 1.00, 0.83, 0.67, 0.57, and 0.50. For studying the automatic matching accuracy, the phantom images were acquired with a six‐dimensional shifting table. The image quality and correction of automatic matching were compared. With a decreasing total mAs ratio, the noise of the images increased and the low‐contrast resolution decreased, while the accuracy of the automatic matching did not change. Therefore, this study shows that a change of the total mAs while acquiring CBCT images has no effect on the automatic matching of Catphan 504 phantom in XI system.

PACS number(s): 87.57.C‐, 87.57.Q‐

## I. INTRODUCTION

The goal of radiation therapy is to deliver an accurate radiation dose to the target while minimizing unnecessary dose in the surrounding normal organs. The complex radiation therapy techniques, such as intensity‐modulated radiation therapy (IMRT), image‐guided radiation therapy (IGRT), and stereotactic body radiation therapy (SBRT), have been used to improve the efficiency of radiation treatment.^1^, ^2^, ^3^, ^4^ Movement of the tumor and the organs or mechanical problems of linear accelerator can affect the treatment outcome during treatment. Previous studies have been reported to minimize such errors. Bloemen‐van Gurp et al.^(5)^ reported accurate tumor positioning in SBRT using three‐dimensional ultrasound imaging and active breathing control as an image guidance tool.

In order to reduce errors, the anatomy of the patient was generally checked during the course of treatment. The verification of the treatment position was performed with a film or electronic portal imaging device (EPID), or with kV X‐ray systems such as the X‐ray Volumetric Imager (XVI, Elekta Oncology Systems, Norcross, GA) and On‐Board Imager (OBI, Varian Medical Systems, Palo Alto, CA).^6^, ^7^ In particular, kV cone‐beam computed tomography (CBCT) has been commonly used to verify the patient setup in order to improve radiation treatment accuracy.

Besides, CBCT can be used to evaluate the three‐dimensional volumetric image information for the anatomy of the patient.^8^, ^9^

According to Task Group 57 of the American Association of Physicist in Medicine (AAPM),^(10)^ a patient receives an additional radiation dose of approximately 15–20 mGy from the daily CBCT imaging for the treatment position matching in a prostate cancer case. Palm et al.^(11)^ reported that the additional dose was 11.6 and 34.2 mGy for a chest and pelvis case, respectively. The CBCT image acquisition can increase the risk of unnecessary radiation dose delivered to patients. Islam et al.^(12)^ reported that additional doses from 1.6 cGy (at the center) to 2.3 cGy (at the surface) were delivered to patients during the CBCT image acquisition with a cylindrical‐shaped phantom for a typical full‐rotation imaging protocol (with 120 kVp beams, a field of view of 26×26cm, and total technique settings of 660 mAs). Wen et al.^(13)^ reported that absolute doses for pelvic CBCT scans were measured to be from 2 to 5 cGy at the skin, with a maximum of 11 cGy at the femoral bone, using thermoluminescence dosimeter (TLD) of a RANDO pelvic heterogeneous phantom. Although the additional dose is small compared to the treatment dose, it may increase the stochastic effects of radiation and occurrence probability of a secondary malignant cancer. Therefore, it is necessary to minimize the additional dose. The image quality for the treatment position check is important, but the irradiated dose to patients should be considered carefully.

In this study, we evaluate the accuracy of automatic matching of a phantom in CBCT images relative to the reduction of total tube current–exposure time product (mAs) for verifying the treatment position.

## II. MATERIALS AND METHODS

An X‐ray imaging (XI) system (Version 2.0, Varian Medical Systems) with a Varian Truebeam STx linear accelerator (TBX Linac, Version 2.0.33.2) and the Catphan 504 phantom (The Phantom Laboratory, Salem, NY) were used for this study. The kV image system of XI system consists of a kV X‐ray generator with a kV imager mounted orthogonally to the gantry axis using a robotic arm. The Catphan 504 phantom has a design of several modules and is made to measure various image quality indices, such as sensitometry, slice thickness, spatial resolution, contrast, and uniformity.

**Figure 1:**
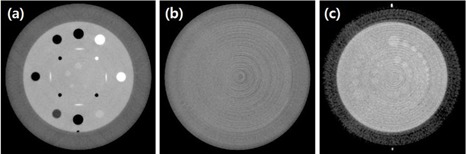
kV CBCT images of Catphan 504 phantom: (a) the sensitometry module, (b) the uniformity module, and (c) the low‐contrast module.

In the XI system of TBX Linac, kV CBCT images can be obtained in six modes: “Head,” “ImageGently,” “Pelvis,” “Pelvis Obese,” “Spotlight,” and “Thorax.” For this study, we used only three modes: “Head,” “Thorax,” and “Pelvis.” For the Head mode, the images were acquired using the full‐fan scan with a full bowtie filter and a 200° scan arc. The reconstruction field of view is $25\times 25\;{\text{cm}}^{2}$ and the reconstruction length is 17 cm. The images under the Thorax and Pelvis modes were acquired using the half‐fan scan (a reconstruction field of view of $46\times 46\;{\text{cm}}^{2}$ and a reconstruction length of 15.5 cm) with a half bowtie filter and a 360° scan arc. The tube voltage was fixed for each acquisition mode. However, the total mAs ratio could be varied from 0.83 to 0.50. CBCT scan was repeated three times. Table 1 shows CBCT setting parameters used for acquisition mode of each image in the XI system. All scans were saved and exported as Digital Imaging and Communications in Medicine (DICOM) files under the advanced reconstruction mode of TBX Linac. The Catphan QA automated computer analysis program (http://catphanqa.imageowl.com) was used to analyze these files. Then the Hounsfield units (HUs), as well as the noise and low‐contrast resolution of images, were evaluated varying total mAs (Fig. 1).

The HU was measured with the sensitometry module of the phantom. The module included eight materials with known densities, such as air, polymethylpentene (PMP), low‐density polyethylene (LDPE), water, polystyrene, acrylic, delrin, and Teflon. The HU was measured in the center of materials.

**Table 1:** Cone‐beam computed tomography setting parameters used for image acquisition mode in the X‐ray imaging system

	*Ratio of Total Tube Current–exposure Time Product*				
*Protocol*	*1.00*	*0.83*	*0.67*	*0.57*	*0.50*
*Head*					
kVp	100	100	100	100	100
Total mAs	150	125	100	87.5	75
CTDIw (cGy)	0.29	0.25	0.19	0.18	0.15
*Thorax*					
kVp	125	125	125	125	125
Total mAs	270	225	180	157.5	135
CTDIw (cGy)	0.36	0.30	0.23	0.21	0.18
*Pelvis*					
kVp	125	125	125	125	125
Total mAs	1080	900	720	630	540
CTDIw (cGy)	1.43	1.19	0.94	0.84	0.71

CTDIw=weighted computed tomography dose index.

The noise was measured with the uniformity module of the phantom. The noise represents the statistical fluctuations in the density of X‐ray photons that are attenuated after passing through X‐ray objects. The noise was calculated as the standard deviation of HU values from a center region‐of‐interest (ROI) with the uniformity module.

Furthermore, the low‐contrast resolution was measured in the low‐contrast module of the phantom. The low‐contrast module consisted of supraslice and subslice contrast targets. The supraslice contrast targets had three nominal target contrast levels of 0.3%, 0.5%, and 1.0%. There were nine supraslice targets with diameters of 2, 3, 4, 5, 6, 7, 8, 9, and 15 mm. The low‐contrast value represents the diameter of the smallest detectable target for each contrast value, and is based on the nominal contrast, estimation of noise, and detection level. In this study, we analyzed the 1.0% target contrast level of the supraslice contrast targets.

In addition, we evaluated the accuracy of the automatic matching of phantom position for the mAs changes in kV CBCT. Automatic matching is an objective evaluation where the matching is automatically corrected using the computer software. The phantom was positioned at the end of the Varian PerfectPitch six‐degrees‐of‐freedom couch with the phantom case supplied by the manufacturer. The center of the phantom was aligned in the center of the gantry rotation. After setting up the phantom, the table was moved 0.2 cm along the vertical, lateral, and longitudinal directions, and was rotated 0.5° along the roll, pitch, and yaw axes. The reference displacement of the table shift was $1.345\;\text{cm}(|{\overset{ˉ}{\Delta }}_{\text{Ref}}|=1.345\;\text{cm})$. CBCT images of the phantom for the shifting table were acquired the Head, Thorax, and Pelvis modes with varying the total mAs. In the sensitometry module, an ROI of the automatic matching was set in the acquired image at an intensity range of HU value from ‐200 to 800. Then the shifted values of the table were compared with the actual moved values and auto‐matched values.

Statistical analyses were performed using Statistical Package for the Social Sciences (SPSS, version 20.0, SPSS Inc., Chicago, IL). Kruskal‐Wallis test was done on the mean HU value, noise, and shift of the couch for total mAs changes. The significance level was less than 0.05.

## III. RESULTS

### A. Image quality

Table 2 lists the average HU value of the sensitometry targets in the sensitometry module and the mean values of the noise in the uniformity module for the Catphan 504 phantom while changing the total mAs.

With varying total mAs (p<0.05), the HU values of Delrin (p=0.009) and Teflon (p=0.010) showed statistically significant differences in the Head mode; in the Thorax mode, the HU values of Acrylic (p=0.045), Delrin (p=0.017), and Teflon (p=0.009) showed statistically significant differences; in the Pelvis mode, only the HU value of Teflon (p=0.017) showed a significant difference. However, the other targets did not exhibit significant differences in HU values with changing the total mAs (p>0.05).

**Table 2:** The averaged HU value measured at various targets using sensitometry module and the mean measured noise using uniformity module for the Catphan 504 phantom with varying total mAs ratios

		*Ratio of Total Tube Current–exposure Time Product*					
*Material*	*HU Range*	*1.00*	*0.83*	*0.67*	*0.57*	*0.50*	*p‐value*
*Head*							
Air	‐1046:‐986	‐1000.0±0.0	‐1000.0±0.0	‐1000.0±0.0	‐1000.0±0.0	‐1000.0±0.0	1.000
PMP	‐220:‐172	‐180.7±4.0	‐189.7±2.3	‐190.3±6.0	‐187.3±4.5	‐195.3±3.1	0.064
LDPE	‐121:‐87	‐99.3±4.2	‐100.3±1.5	‐101.7±7.1	‐104.7±5.8	‐106.7±6.7	0.470
Polystyrene	‐65:‐29	‐32.7±2.1	‐29.0±3.0	‐29.3±2.3	‐33.0±1.7	‐29.3±9.0	0.421
Acrylic	92:137	139±1.7	145.3±0.6	152.7±4.2	148.3±6.5	148.0±4.0	0.053
Delrin	344:387	406.7±0.6	414.0±1.7	426.0±2.6	431.7±1.2	445.0±3.6	0.009
Teflon	941:1060	1058.7±5.7	1065.0±1.7	1100.0±7.2	1119.0±1.0	1143.0±4.4	0.010
Noise	‐	32.0±1.3	34.5±0.9	40.8±1.3	42.8±1.0	47.8±0.6	0.009
*Thorax*							
Air	‐1046:‐986	‐1000.0±0.0	‐1000.0±0.1	‐1000.0±0.2	‐1000.0±0.3	‐1000.0±0.4	1.000
PMP	‐220:‐172	‐190.7±2.3	‐185.3±1.2	‐185.7±1.5	‐186.0±2.0	‐184.0±4.0	0.151
LDPE	‐121:‐87	‐93.0±2.6	‐91.7±2.1	‐92.0±4.4	‐90.0±6.9	‐88.7±2.3	0.497
Polystyrene	‐65:‐29	‐32.7±3.5	‐31.0±3.6	‐29.3±2.3	‐28.7±3.1	‐25.3±1.2	0.072
Acrylic	92:137	136.0±6.1	137.0±1.7	142.7±4.6	143.0±4.4	150.0±3.5	0.045
Delrin	344:387	377.7±2.5	382.3±4.7	393.0±5.2	390.7±6.0	405.0±3.0	0.017
Teflon	941:1060	1007.3±3.1	1014.3±2.1	1037.3±4.2	1050.0±4.4	1060.3±3.2	0.009
Noise	‐	16.1±0.4	16.1±0.4	18.3±0.3	18.8±0.6	19.9±0.4	0.022
*Pelvis*							
Air	‐1046:‐986	‐1000.0±0.0	‐1000.0±0.1	‐1000.0±0.2	‐1000.0±0.3	‐1000.0±0.4	1.000
PMP	‐220:‐172	‐203.7±4.0	‐187.7±2.1	‐188.7±0.6	‐189.0±1.7	‐188.0±1.7	0.091
LDPE	‐121:‐87	‐112.0±1.7	‐96.3±0.6	‐96.7±2.1	‐94.3±2.5	‐94.7±3.1	0.088
Polystyrene	‐65:‐29	‐53.7±0.6	‐37.0±1.0	‐38.0±1.7	‐37.0±2.6	‐35.3±3.1	0.093
Acrylic	92:137	109.7±3.5	126.0±2.6	129.3±1.5	127.3±1.5	129.7±3.2	0.058
Delrin	344:387	347.7±4.0	367.0±6.1	369.0±6.2	369.0±4.6	373.3±3.1	0.074
Teflon	941:1060	947.3±3.1	978.7±1.5	980.3±1.5	983.7±2.1	985.3±4.2	0.017
Noise	‐	9.5±0.1	11.2±0.1	12.7±1.5	13.5±1.5	13.1±1.4	0.024

Units are in HUs.

PMP=polymethylpentene; LDPE=low‐density polyethylene; Noise=image noise; HU=Hounsfield units.

**Figure 2:**
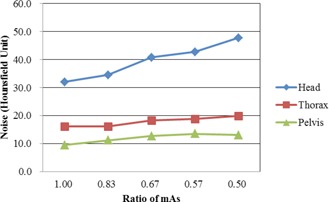
The mean values of the noises in the Catphan 504 with varying total mAs ratios.

The mean measured noises with a total mAs ratio of 1.00 are 32.0±1.3,16.1±0.4, and 9.5±0.1 HU, for the Head, Thorax, and Pelvis mode, respectively (p<0.05). The mean measured noises with a total mAs ratio of 0.50 are 47.8±0.6,19.9±0.4, and 13.1±1.4 HU for the Head, Thorax, and Pelvis mode, respectively (p<0.05). Figure 2 shows the mean values of the noises.

Table 3 shows the low‐contrast resolution in the low‐contrast module with varying total mAs. With a the total mAs ratio of 1.00, the visible diameter of the supraslice contrast targets (under 1.0% target contrast level) was from 6 to 8 mm in the Thorax mode, and from 3 to 5 mm in the Pelvis mode; with a total mAs ratio of 0.5, the visible diameter was from 8 to 9 mm in the Thorax mode, and from 5 to 8 mm in the Pelvis mode. However, the visible diameter in the Head mode was more than 15 mm for all total mAs ratios.

**Table 3:** The low‐contrast resolution in the low‐contrast module for the Catphan 504 phantom with varying total mAs ratios

	*Ratio of Total Tube Current–exposure Time Product*			
*CBCT Mode*	*1.00*	*0.83*	*0.67*	*0.57*	*0.50*
*Head*					
#1	>15	>15	>15	>15	>15
#2	>15	>15	>15	>15	>15
#3	>15	>15	>15	>15	>15
*Thorax*					
#1	7	7	9	9	9
#2	6	9	7	7	9
#3	8	7	9	9	8
*Pelvis*					
#1	3	4	4	7	5
#2	5	8	7	5	8
#3	4	4	5	7	5

units=mm; #1, #2, and #3=the number of kV CBCT scans.

### B. Accuracy of automatic matching

Table 4 shows the results of automatic matching for phantom position with varying total mAs in CBCT. The difference between the corrected and actual shifted values along the roll, pitch, and yaw axes was within 0.1°. The maximum auto‐matched displacement of the table was 1.345±0.008cm and the minimum value was 1.319±0.002cm. However, there is no statistically significant difference in the changed angle and table displacement with varying total mAs (p>0.05).

**Table 4:** The results of automatic matching for the Catphan 504 phantom position with varying total mAs ratios in the X‐ray imaging system

	*Ratio of Total Tube Current–exposure Time Product*					
*Couch*	*1.00*	*0.83*	*0.67*	*0.57*	*0.50*	*p‐value*
*Head*						
Yaw (°)	‐0.07±0.06	‐0.07±0.06	0.00±0.00	0.00±0.00	0.00±0.00	0.520
Pitch (°)	0.00±0.10	0.00±0.01	0.00±0.01	‐0.01±0.01	‐0.01±0.01	0.645
Roll (°)	‐0.10±0.00	‐0.01±0.01	0.00±0.01	‐0.01±0.01	0.00±0.01	0.406
$|{\overset{ˉ}{\Delta }}_{\text{shift}}|\;(\text{cm})$	1.345±0.008	1.340±0.006	1.336±0.008	1.336±0.007	1.333±0.002	0.478
$|{\overset{ˉ}{\Delta }}_{\text{shift}}|$ ‐ $|{\overset{ˉ}{\Delta }}_{\text{Ref}}|\;(\text{cm})$	0.000±0.008	‐0.005±0.006	‐0.009±0.008	‐0.009±0.007	‐0.012±0.002	‐
*Thorax*						
Yaw (°)	0.10±0.00	0.10±0.00	0.10±0.00	0.07±0.06	0.07±0.06	0.520
Pitch (°)	0.03±0.06	0.07±0.06	0.07±0.06	0.03±0.06	0.10±0.00	0.458
Roll (°)	0.00±0.00	0.00±0.00	0.00±0.00	‐0.03±0.06	0.00±0.00	0.406
$|{\overset{ˉ}{\Delta }}_{\text{shift}}|\;(\text{cm})$	1.327±0.003	1.322±0.002	1.322±0.005	1.320±0.002	1.319±0.002	0.081
$|{\overset{ˉ}{\Delta }}_{\text{shift}}|‐|{\overset{ˉ}{\Delta }}_{\text{Ref}}|\;(\text{cm})$	‐0.018±0.003	‐0.023±0.002	‐0.023±0.005	‐0.025±0.002	‐0.026±0.002	‐
*Pelvis*						
Yaw (°)	‐0.07±0.06	‐0.07±0.06	0.03±0.06	0.03±0.06	0.00±0.00	0.104
Pitch (°)	0.00±0.10	0.07±0.06	0.07±0.06	0.00±0.00	0.07±0.06	0.406
Roll (°)	‐0.07±0.06	‐0.03±0.06	0.07±0.06	0.03±0.06	0.07±0.06	0.097
$|{\overset{ˉ}{\Delta }}_{\text{shift}}|\;(\text{cm})$	1.329±0.002	1.329±0.002	1.332±0.004	1.330±0.004	1.333±0.002	0.608
$|{\overset{ˉ}{\Delta }}_{\text{shift}}|‐|{\overset{ˉ}{\Delta }}_{\text{Ref}}|\;(\text{cm})$	‐0.016±0.002	‐0.016±0.002	‐0.013±0.004	‐0.015±0.004	‐0.012±0.002	‐

Angle (°) is difference of given couch angle coordinates in OBIs (On‐Board Imager system) with phantom's setup position.

$|{\overset{ˉ}{\Delta }}_{\text{shift}}|$ is auto‐matched displacement of given couch shift (X: vertical, Y: longitudinal, Z: lateral) coordinates in OBIs ($|{\overset{ˉ}{\Delta }}_{\text{shift}}|=(X,Y,Z)=\sqrt{X^{2}+Y^{2}+Z^{2})}$ and $|{\overset{ˉ}{\Delta }}_{\text{Ref}}|$ is reference displacement of actual moved phantom from setup position ($|{\overset{ˉ}{\Delta }}_{\text{Ref}}|=1.345$).

$|{\overset{ˉ}{\Delta }}_{\text{shift}}|‐|{\overset{ˉ}{\Delta }}_{\text{Ref}}|\;(\text{cm})$ is disagreement between the auto‐matched values and the actual moved values.

## IV. DISCUSSION

The aim of this study is to evaluate the accuracy of the auto matching relative to the quality change of CBCT images for various total mAs ratios. CBCT image acquisition can increase a radiation dose delivered to patients. Some previous studies have been performed to reduce the additional radiation dose delivered to patients. Islam et al.^(12)^ reported that the additional dose could be reduced by employing lower‐energy kVp beams and smaller field of view, or using half‐rotation scans. Besides, Ding et al.^(14)^ reported that the dose could be minimized by optimizing the scan length, exposure setting, gantry rotation angle selection, and full‐fan bowtie whenever possible through Monte Carlo analyses for kV CBCT scans.

One way to reduce the delivered radiation dose to patients during the CBCT image acquisition is to decrease the mAs. Song et al.^(15)^ mentioned that the doses increased linearly with the increase of the mAs setting and the increased doses (mAs) could improve the image quality. According to the studies by Tomic et al.^(16)^ and Hu and McLean^(17)^, the absorbed radiation dose was reduced in the skin and tissue when using at lower mAs on the same CBCT protocol. As shown in Table 1, the weighted computed tomography dose index (CTDIw) provided by XI system decreased with decreasing the total mAs ratio.

However, the image quality was downgraded due to the excessive quantum noise produced under the condition of a low mAs protocol.^(18)^ Kamath et al.^(19)^ reported that the noise was observed to asymptotically decrease with decreasing mAs, while most image quality parameters showed improvement with increasing mAs. In other words, the CBCT image quality decreased with decreasing mAs, which is consistent with our results. In this work, we assessed the accuracy of the image automatic matching for the varying image quality with changing total mAs. Our results indicated that the noise of the image increased and the low‐contrast resolution decreased with decreasing mAs, although the HU value was relatively stable. The XI system of Varian has a function to correct the auto‐matched images on the basis of the HU value. In this study, the image automatic matching was used to objectively compare the kV CBCT images and the CT simulation images. The corrected images using the automatic matching did not differ with varying mAs.

Overall, these results indicate that the kV CBCT image acquired by reducing the total mAs has a lower image quality, while the accuracy of the image is maintained by automatic matching. Furthermore, lower mAs can reduce the additional radiation dose delivered to patients.

This study has certain limitations. Since the analysis used a phantom, it is difficult to generalize the findings of this research to patients. Moreover, this study did not analyze the spatial resolution. The spatial resolution is important in automatic matching using CBCT images, but there is a moderate correlation between the mAs and the spatial resolution.^(20)^ However, regarding the justification and optimization presented in International Commission on Radiological Protection (ICRP), the mAs of CBCT is recommended to be adjusted downward for correcting the treatment position in radiation therapy. Meanwhile, a phase study on changes in the body weight or age of a patient is considered necessary. In the future, we will add humanoid phantom study to investigate the optimal mAs value and tube voltage which required in CBCT imaging among various treatment sites of patients.

The change in kV CBCT image quality with varying total mAs was evaluated on an XI system with the Catphan 504 phantom. The accuracy of image correction using automatic matching was assessed. The noise of the image increased and the low‐contrast resolution decreased with decreasing mAs. However, no change was observed in the accuracy of the auto‐matched images with varying the total mAs. Therefore, this study shows that a change of the total mAs while acquiring CBCT images has no effect on the automatic matching of Catphan 504 phantom in XI system.

## COPYRIGHT

This work is licensed under a Creative Commons Attribution 3.0 Unported License.
